# Androgen Receptor Antagonists and Anti-Prostate Cancer Activities of Some Newly Synthesized Substituted Fused Pyrazolo-, Triazolo- and Thiazolo-Pyrimidine Derivatives

**DOI:** 10.3390/ijms151121587

**Published:** 2014-11-24

**Authors:** Saleh A. Bahashwan, Ahmed A. Fayed, Mohamed A. Ramadan, Abd El-Galil E. Amr, Naif O. Al-Harbi

**Affiliations:** 1 Pharmacology and Toxicology Department, College of Pharmacy, Taibah University, Almadinah Almunawarah 22624, Saudi Arabia; E-Mail: drsalehamb@yahoo.com; 2 Respiratory Therapy Department, College of Medical Rehabilitation Sciences, Taibah University, Almadina Almanoara 22624, Saudi Arabia; 3 National Research Center, Cairo, Dokki 12622, Egypt; E-Mail: aeamr1963@yahoo.com; 4 Microbiology and Immunology Departments, College of Medicine, Taibah University, Almadinah Almunawarah 22624, Saudi Arabia; E-Mail: mmostafa201120@yahoo.com.; 5 Pharmaceutical Chemistry Department, Drug Exploration and Development Chair (DEDC), College of Pharmacy, King Saud University, Riyadh 11451, Saudi Arabia; 6 Pharmacology and Toxicology Department, College of Pharmacy, King Saud University, Riyadh 11451, Saudi Arabia; E-Mail: ouf.nabil@yahoo.com.

**Keywords:** naphthalinothiazolohydrazine, pyrazolopyrimidine, thiazolopyrimidine, anticancer activities

## Abstract

A series of substituted pyrazole, triazole and thiazole derivatives (**2**–**13**) were synthesized from 1-(naphtho[1,2-*d*]thiazol-2-yl)hydrazine as starting material and evaluated as androgen receptor antagonists and anti-prostate cancer agents. The newly synthesized compounds showed potent androgen receptor antagonists and anti-prostate cancer activities with low toxicity (lethal dose 50 (LD_50_)) comparable to Bicalutamide as reference drug. The structures of newly synthesized compounds were confirmed by IR, ^1^H-NMR, ^13^C-NMR, and MS spectral data and elemental analysis. The detailed synthesis, spectroscopic data, LD_50_ values and pharmacological activities of the synthesized compounds are reported.

## 1. Introduction

Cancer poses a serious human health problem despite much progress in understanding its biology and pharmacology. Consequently, the design of new structures employed as antitumor agents is one of the most urgent research areas in contemporary medicinal chemistry. Some new heterocyclic compounds containing a thiazole ring were synthesized and used as antibacterial, antifungal [1,2] and anti-inflammatory agents (chemokine-receptor antagonists) [3]. These derivatives are also well known for their pronounced inhibition of the growth of cytomegalovirus (HCMV)-human cytomegalovirus [4], and are used as corticotrophin-releasing hormone (CRH-R1) receptor antagonists (display antidepressant activity) [5]. Thiazolopyrimidine derivatives were studied as potential drug candidates with anticancer activities [6,7]. In a previous work, we reported that certain of our newly substituted heterocyclic compounds exhibited antitumor activities [8,9,10]. Pyrimidine has gained considerable attention because of its diversity in biological activity and widespread applications in pharmaceuticals fields [11,12]. For examples, pyrimidine derivatives are used as Tie-2 kinase inhibitors [13,14], Human immunodeficiency virus type-1 (HIV-1) inhibitors [15,16], antimalarials [17,18], adenosine A1 receptor antagonists [19], anticancer agents [20], analgesics [21], cardiovascular [22] and anti-allergic agents [23,24]. The potent biological activities of various vitamins and drugs [25,26,27,28] are primarily contributed by the presence of heterocyclic rings in their molecular make-up. In view of these reports and in continuation of our previous works in heterocyclic chemistry, we report here newly synthesized substituted pyrazole derivatives. The androgen receptor antagonist, and anti-prostate cancer activities for the synthesized compounds was evaluated using Bicalutamide as a reference drug.

## 2. Results and Discussion

### 2.1. Chemistry

**Scheme 1:**
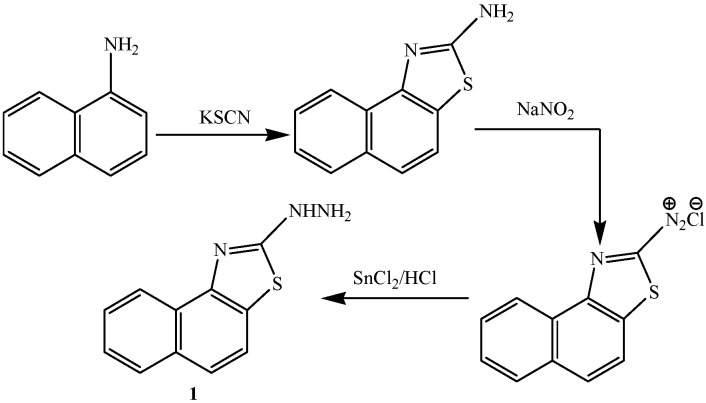
Synthetic route of 1-(naphtho[1,2-*d*]thiazol-2-yl)hydrazine **1**.

In the present study, a series of substituted pyrazole, triazole and thiazole derivatives (**2**–**13**) were synthesized from 1-(naphtho[1,2-*d*]thiazol-2-yl)hydrazine (**1**) as starting material. The starting material **1** was synthesized according to the reported literature [29] (Scheme 1).

**Scheme 2:**
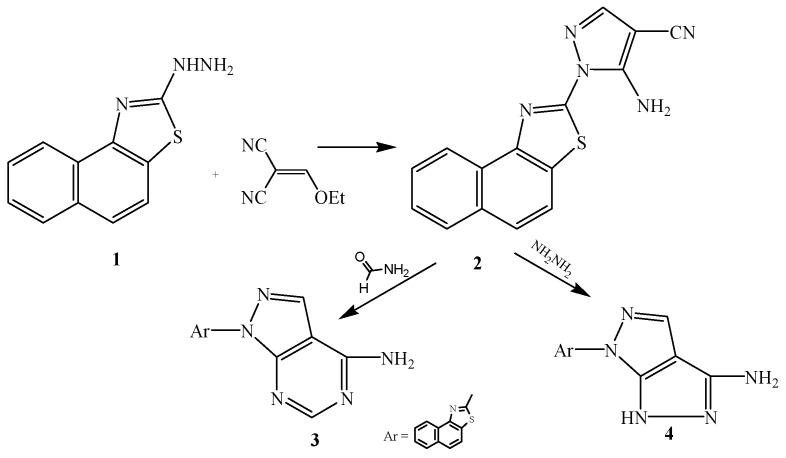
Synthetic route of compounds **2**–**4**.

Treatment of **1** with ethoxymethylene malomonitrile afforded the corresponding 3-amino-4-cyanopyrazolo derivatives **2**, which was reacted with formamide or hydrazine hydrate to afford the corresponding 3-aminopyrazolopyrimidine **3** and aminopyrazolopyrazole derivative **4**, respectively (Scheme 2).

**Scheme 3:**
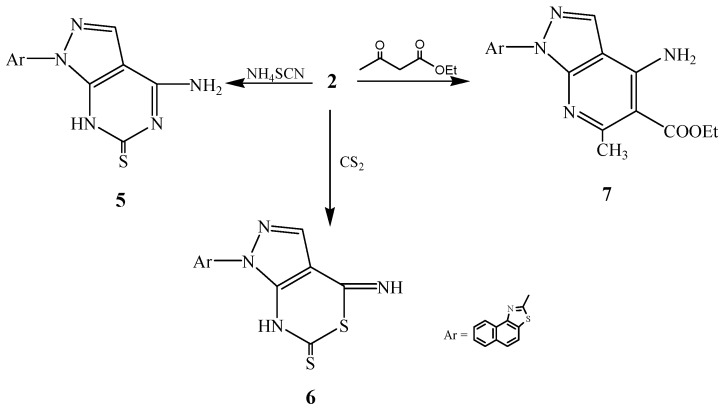
Synthetic route of compounds **5**–**7**.

Compound **2** was reacted with ammonium isothiocyanate in the presence of ethanol to afford the corresponding pyrazolopyrimidine derivative **5**. Also, compound **2** was treated with carbon disulphide or ethyl acetoacetate to afford the corresponding pyrazolothiazolino derivative **6** pyrazolopyridino derivative **7**, respectively (Scheme 3).

**Scheme 4:**
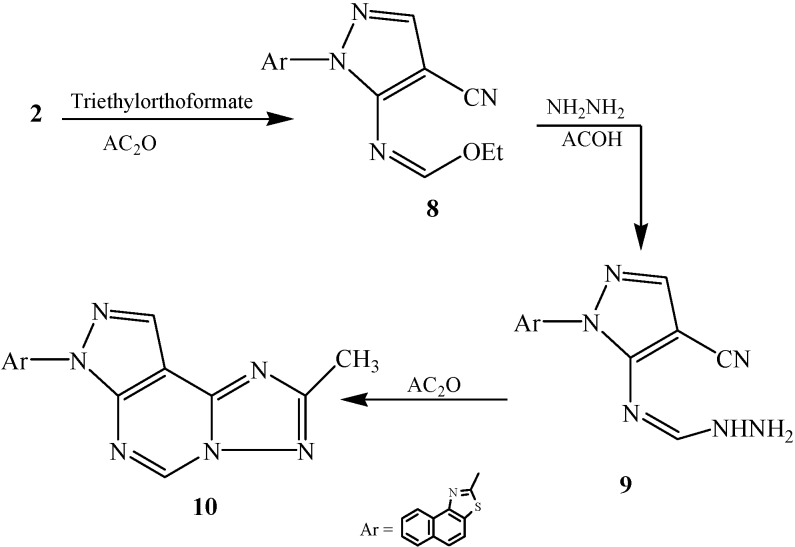
Synthetic route of compounds **8**–**10**.

Additionally, compound **2** was reacted with triethylorthoformate in the presence of acetic anhydride to afford compound **8**, which was reacted with hydrazine hydrate in the presence of acetic acid to afford the hydrazine derivative **9**. The latter compound **9** was cyclized by acetic anhydride to the pyrazolotriazolopyrimidine derivative **10** (Scheme 4).

**Scheme 5:**
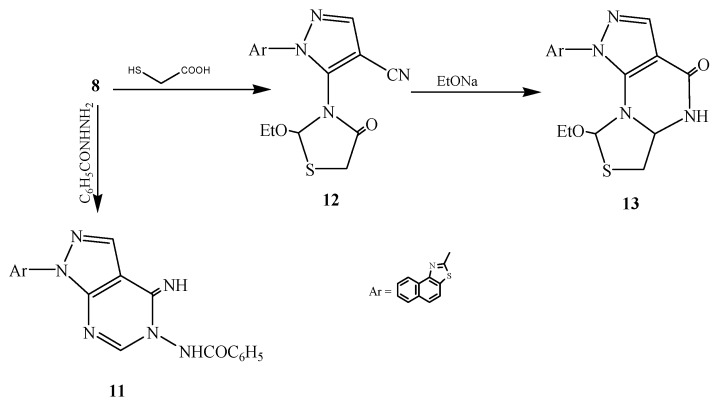
Synthetic route of compounds **11**–**13**.

Finally, compound **8** was reacted with benzohydrazide or mercapto acetic acid to afford the corresponding compounds **11** and **12**, respectively. Compound **12** was cyclized by refluxing with ethanolic sodium ethoxide to pyrazolothiazolopyrimidine derivative **13** (Scheme 5).

### 2.2. Pharmacological Evaluation

All the candidates were evaluated for their *in vitro* androgen receptor (AR) antagonistic activities using a reporter assay, and the resulting inhibitory concentration (IC_50_) values are listed in Table 1. All new compounds were found to have potent *in vitro* AR antagonistic activities compared to Bicalutamide, but, compound **1** was devoid from any activities. Also, evaluated was the *in vivo* anti-androgenic activity in castrated immature rats, and the resulting percent inhibition values are listed in Table 2. The tested compounds were found to have potent *in vivo* anti-androgenic activities compared to Bicalutamide except compound **1** which was devoid from any activities.

In addition, all the analogues were evaluated by anti-prostate cancer screening anti-androgenic bioassay in human prostate cancer cells; the resulting IC_50_ values are listed in Table 3. All tested newly synthesized compounds were found to have potent anti-prostate cancer activities compared to Bicalutamide except compound **1** which was devoid from any activities. The target compounds were tested for cytotoxicity against two human prostate cancer cell lines, lymph node carcinoma of the prostate (LNCaP) and human prostate cancer cell lines (PC-3). The LNCaP cell line is an androgen-dependent human prostate cancer cell line that expresses mutant AR, and the PC-3 cell line is an androgen-independent human prostate cancer cell line that does not express functional AR. All the tested compounds exhibited significant cytotoxicity in either LNCaP or PC-3 cells.

**Table 1:** *In vitro* androgen receptor antagonistic activities of the newly synthesized compounds.

Compound No.	IC_50_ (µM)
Bicalutamide	0.89
**1**	Inactive
**2**	0.031
**3**	0.037
**4**	0.034
**5**	0.041
**6**	0.044
**7**	0.057
**8**	0.077
**9**	0.075
**10**	0.023
**11**	0.039
**12**	0.091
**13**	0.029

**Table 2:** *In vivo* anti-androgen activities of the newly synthesized compounds **1**–**13**.

Compound No.	% (Inhibition)	ED_50_ (mg/kg)
Bicalutamide	75	1.6
**1**	Inactive	Inactive
**2**	95.49	0.049
**3**	91	0.063
**4**	93.98	0.051
**5**	89.09	0.089
**6**	88.76	0.092
**7**	87.56	0.094
**8**	85.43	0.097
**9**	86.56	0.095
**10**	98.87	0.071
**11**	90.01	0.045
**12**	84.65	0.098
**13**	97.56	0.047

**Table 3:** Anti-prostate cancer activities of the newly synthesized compounds **1**–**13**.

Compound No.	IC_50_ (nM) PC-3	IC_50_ (nM) LNCaP
Bicalutamide	822	619.00
**1**	Inactive	Inactive
**2**	156.78	51.47
**3**	177.45	58.58
**4**	178.6	56.59
**5**	190.1	64.28
**6**	201.23	72.39
**7**	256.78	74.56
**8**	278.67	78.54
**9**	268.77	76.65
**10**	109.29	43.54
**11**	180.01	61.58
**12**	288.78	79.98
**13**	145.67	49.65

### 2.3. Determination of Acute Toxicity (Lethal Dose 50 (LD_50_))

The LD_50_ was determined by using rats injected with different increasing doses of the synthesized compounds. The dose that killed 50% of the animals was calculated according to Austen and Brocklehurst [30] (Table 4).

**Table 4:** Acute toxicity (LD_50_) of the synthesized of the newly synthesized compounds **1**–**13**.

Compound No.	LD_50_ (mg/kg)
**1**	1164.45
**2**	163.46
**3**	174.56
**4**	265.67
**5**	266.58
**6**	257.79
**7**	146.80
**8**	235.99
**9**	124.89
**10**	315.78
**11**	324.57
**12**	234.46
**13**	443.35

## 3. Experimental Section

### 3.1. General Experimental Procedures

Melting points were determined on open glass capillaries using an Electrothermal IA 9000 SERIES digital melting point apparatus (Electrothermal, Essex, UK), and are uncorrected. Elemental analyses were performed with all final compounds on an Elementar, Vario EL, Microanalytical Unit, National Research Centre, Cairo Egypt, and the results were found within *ca.* 0.4% of the theoretical values. Analytical data were obtained from the Microanalytical Unit, Cairo University, Egypt. The IR spectra (KBr) were recorded on a FT IR-8201 PC spectrophotometer (Shimadzu, Tokyo, Japan). The ^1^H- and ^13^C-NMR spectra were measured with a JEOL FTGNMEX 270, 270 MHz instrument (JEOL, Tokyo, Japan) in DMSO-*d*_6_, and chemical shifts were recorded in (δ, ppm) relative to tetramethylsilane (TMS). The mass spectra were run at 70 eV with a Finnigan SSQ 7000 spectrometer (Thermo Electron Corporation, Madison, WI, USA.) using EI, and the values of *m*/*z* are indicated in Dalton. TLC (Silica gel, aluminum sheets 60F_254_, Merck, Darmstadt, Germany) followed the reactions.

### 3.2. Synthetic Procedures

#### 3.2.1. Synthesis of 2-[3-Amino-4-cyanopyrazol-2-yl]naphthalino[1,2-*d*]thiazole (**2**)

A mixture of compound **1** (0.01 mol) and ethoxymethylene malononitrile (0.01 mol) in absolute ethanol (30 mL) was refluxed for 2 h. The solvent was evaporated under reduced pressure; the obtained solid was crystallized from ethanol to give the title product **2** as yellow powder. Yield: 87%; m.p.: 256–258 °C; IR (KBr, cm^−1^): 3345–3315 (NH_2_) and 2215 (CN) cm^−1^; ^1^H-NMR (DMSO-*d*_6_): δ = 6.78–7.61 (m, 7H, 6Ar–H and 1H_Pyrazolo_) and 10.43 (b, 2H, NH_2_ exchangeable with D_2_O) ppm; ^13^C-NMR (DMSO-*d*_6_): δ = 112.2, 112.2, 113.6, 118.2, 119.4, 127.1, 128.5, 129.1, 134.4, 143.5 (Ar–C), 121.4 (CN), 131.2 (thiazole–C), 132.4, 136.2, 142.3 (pyrazole–C) ppm; MS *m*/*z* (%): 291 (M^+^, 23), 265 (33), 249 (46), 212 (25), 184 (100), 126 (29), 102 (21), 78 (17). Anal. Calcd. for C_15_H_9_N_5_S (291.37): C, 61.82; H, 3.11; N, 24.04; S, 11.02. Found: C, 61.54; H, 2.96; N, 23.81; S, 10.82.

#### 3.2.2. Synthesis of 2-[6-Aminopyrimidino[4,5-*c*]pyrazol-2-yl]naphthalino[1,2-*d*]thiazole (**3**)

A solution of **2** (0.01 mol) in formamide (20 mL) was refluxed for 2 h. After cooling, the precipitated solid product was collected by filtration, dried and crystallized from methanol to give the title product **3** as reddish brown powder. Yield: 66%; m.p.: 290–292 °C; IR (KBr, cm^−1^): 3325 (NH_2_) cm^−1^; ^1^H-NMR (DMSO-*d*_6_): δ = 6.64–7.52 (m, 8H, 6Ar–H, 1H_Pyrazolo_ and 1H_Pyrimidino_) and 11.12 (bs, 2H, NH_2_ exchangeable with D_2_O) ppm; ^13^C-NMR (DMSO-*d*_6_): δ = 112.1, 113.6, 118.2, 119.4, 127.1, 128.5, 129.1, 134.4, 143.5, 145.4 (Ar–C), 142.3, 145.4, 149.1 (pyrazole–C), 154.1 (thiazole–C), 151.4, 151.5 (pyrimidine–C) ppm; MS *m*/*z* (%): 318 (M^+^, 19), 302 (25), 276 (41), 212 (32), 184(47), 126 (100), 102 (37), 78 (14). Anal. Calcd. for C_16_H_10_N_6_S (318.40): C, 60.35; H, 3.16; N, 26.41; S, 10.08. Found: C, 60.14; H, 2.91; N, 26.27; S, 9.86.

#### 3.2.3. Synthesis of 2-[5-Amino-3H-pyrazolo[3,4-*c*]pyrazol-2-yl]naphthalino[1,2-*d*]thiazole (**4**)

A mixture of **2** (0.01 mol) and hydrazine hydrate (0.01 mol) in acetic acid (30 mL) was refluxed for 3 h. The reaction mixture was poured onto ice-cold water. The obtained precipitate solid was filtered off, dried and crystallized from ethanol to give title product **4** as a green powder. Yield 69%; m.p.: > 300 °C; IR (KBr, cm^−1^): 3372–3323 (NH, NH_2_) cm^−1^; ^1^H-NMR (DMSO-*d*_6_): δ = 6.15 (s, 1H, NH exchangeable with D_2_O), 7.21–7.58 (m, 7H, 6Ar–H and 1H_Pyrazolo_) and 11.42 (bs, 2H, NH_2_ exchangeable with D_2_O) ppm; ^13^C-NMR (DMSO-*d*_6_): δ = 111.5, 112.7, 116.2, 118.2, 125.5, 129.9, 131.4, 133.6, 144.4, 146.2 (Ar–C), 143.2, 145.3, 148.3, 152.3 (pyrazole–C), 151.4 (thiazole–C) ppm; MS *m*/*z* (%): 306 (M^+^, 31), 290 (18), 261 (37), 225 (29), 184 (100), 145 (42), 108 (15), 50 (21). Anal. Calcd. for C_15_H_10_N_6_S (306.39): C, 58.79; H, 3.28; N, 27.43; S, 10.47. Found: C, 58.59; H, 3.11; N, 27.22; S, 10.24.

#### 3.2.4. Synthesis of 2-[6-Amino-3H-4-thiopyrimidino[4,5-*c*]pyrazol-2-yl]naphthalino[1,2-*d*]thiazole (**5**)

A mixture of **2** (0.01 mol) and ammonium isothiocyanate (0.01 mol) in dry acetone (30 mL) was refluxed for 2 h with stirring. The reaction mixture was concentrated under reduced pressure, the obtained solid was collected by filtration, dried and crystallized from ethanol to give the title product **5** as yellow crystals. Yield: 63%; m.p.: 243–145 °C; IR (KBr, cm^−1^): 3375–3315 (NH, NH_2_) and 1090 (C=S) cm^−1^; ^1^H-NMR (DMSO-*d*_6_): δ = 6.93–7.28 (m, 7H, 6Ar-H and 1H_Pyrazolo_), 8.42 (s, 1H, NH exchangeable with D_2_O) and 11.16 (bs, 2H, NH_2_ exchangeable with D_2_O) ppm; ^13^C-NMR (DMSO-*d*_6_): δ = 112.2, 114.3, 117.2, 120.4, 127.6, 130.2, 134.8, 137.4, 143.2, 147.2 (Ar–C), 132.5 (C=S), 145.3 (pyrazole–C), 148.6, 149.2, 152.3 (Pyrimidine–C), 150.3 (thiazole–C) ppm; MS *m*/*z* (%): 350 (M^+^, 26), 334 (53), 264 (26), 225 (19), 199(28), 173 (33), 150 (100), 125 (26), 101 (19 ) 75 (21). Anal. Calcd. for C_16_H_10_N_6_S_2_ (350.50): C, 54.82; H, 2.87; N, 23.98; S, 18.31. Found: C, 54.57; H, 2.65; N, 23.64; S, 18.02.

#### 3.2.5. Synthesis of 2-[6-Imino-3H-4-thiothiazino[4,5-*c*]pyrazol-2-yl]naphthalino[1,2-*d*]thiazole (**6**)

To compound **2** (0.01 mol), carbon disulfide (15 mL) was added drop-wise at room temperature. The reaction mixture was refluxed for 2 h; after cooling, the obtained solid was filtered off, and crystallized from methanol to give the title product **6** as yellow powder. Yield: 59%; m.p.: 264–266 °C; IR (KBr, cm^−1^): 3345–3290 (2 NH) and 1110 (C=S) cm^−1^; ^1^H-NMR (DMSO-*d*_6_): δ = 5.62 (s, 1H, NH exchangeable with D_2_O), 7.11–7.59 (m, 7H, 6Ar–H and 1H_Pyrazolo_) and 9.16 (s, 1H, NH exchangeable with D_2_O) ppm; ^13^C-NMR (DMSO-*d*_6_): δ = 113.3, 114.9, 116.7, 119.1, 126.5, 131.5, 135.3, 136.5, 142.6, 145.6 (Ar–C), 131.2 (C=S), 146.4 (pyrazole–C), 145.3, 148.4, 150.1 (thiazine–C), 152.7 (thiazole–C) ppm; MS *m*/*z* (%): 367 (M^+^, 35), 340 (27), 308 (51), 249 (33), 225 (21), 167 (100), 126 (46), 102 (18), 89 (26) 76 (20). Anal. Calcd. for C_16_H_9_N_5_S_3_ (367.58): C, 52.27; H, 2.46; N, 19.05; S, 26.19. Found: C, 52.08; H, 2.22; N, 18.83; S, 26.02.

#### 3.2.6. Synthesis of 2-[6-Amino-5-ethoxycarbonyl-4-methylpyridino[2,3-*c*]pyrazol-2-yl]naphthalino-[1,2-*d*]thiazole (**7**)

A mixture of **2** (0.01 mol) and ethyl acetoacetate (0.01 mol) in acetic acid (30 mL) in the presence of few drops of triethylamine was refluxed for 3 h. The reaction mixture was poured into water, the separated solid was collected by filtration, dried and crystallized to give ethanol to give the title product **7** as reddish brown powder. Yield: 78%; m.p.: over 300 °C; IR (KBr, cm^−1^): 3345–3110 (NH_2_) and 1730 (C=O ester) cm^−1^; ^1^H-NMR (DMSO-*d*_6_): δ = 1.92 (s, 3H, CH_3_),2.43 (t, 3H, *J* = 6.05 Hz, CH_3_), 3.26 (q, 2H, *J* = 7.05 Hz, CH_2_), 6.84–7.25 (m, 7H, 6Ar–H and 1H_Pyrazolo_) and 11.26 (bs, 2H, NH_2_ exchangeable with D_2_O) ppm; ^13^C-NMR (DMSO-*d*_6_): δ = 24.2 (CH_3_), 31.4 (CH_3_), 63.4 (CH_2_), 112.1, 113.3, 118.5, 119.6, 120.4, 128.4, 132.3, 134.2, 143.4, 145.3 (Ar–C), 142.3, 144.6, 147.3, 148.9, 151.6 (pyridine–C), 150.1 (pyrazole–C), 152.2 (thiazole–C), 171.3 (C=O) ppm; MS *m*/*z* (%): 403 (M^+^, 19), 372 (28), 343 (36), 299 (41 ), 263 (35), 225 (26), 184(34), 146 (100), 108 (23), 50 (14). Anal. Calcd. for C_21_H_17_N_5_SO_2_ (403.49): C, 62.51; H, 4.24; N, 17.36; S, 7.95. Found: C, 62.28; H, 4.03; N, 17.18; S, 7.76.

#### 3.2.7. Synthesis of 2-[3-Ethylimidoformat-4-cyanopyrazol-2-yl]naphthalino[1,2-*d*]thiazole (**8**)

A mixture of **2** (0.01 mol) and triethylorthoformate (2.50 mL) in acetic anhydride (25 mL) was heated under reflux for 2 h. The reaction mixture was poured onto ice water, the formed solid product was collected by filtration, washed with water, dried and crystallized from ethanol to give the title product **8** as yellow crystals. Yield: 71%; m.p.: 225–227 °C; IR (KBr, cm^−1^): 2218 (CN) cm^−1^; ^1^H-NMR (DMSO-*d*_6_): δ = 1.62 (t, 3H, CH_3_, *J* = 6.05 Hz), 3.45 (q, 2H, CH_2_, *J* = 7.05 Hz), 4.27 (s, 1H, CH=N), and 7.14–7.35 (m, 7H, 6Ar–H and 1H_Pyrazolo_) ppm; ^13^C-NMR (DMSO-*d*_6_): δ = 20.2 (CH_3_), 54.4 (CH_2_), 113.6, 114.3, 116.2, 118.7, 120.5, 126.7, 128.1, 130.5, 133.2, 142.4 (Ar–C), 121.3 (C=N), 123.6 (CN), 134.7 (thiazole–C), 133.5, 137.6, 141.4 (pyrazole–C) ppm; MS *m*/*z* (%): 347 (M^+^, 25), 321 (36), 276 (19), 248 (42), 225 (23), 184 (38), 126 (46), 92 (100) 76 (15). Anal. Calcd. for C_18_H_13_N_5_SO (347.43): C, 62.22; H, 3.76; N, 20.16; S, 9.23. Found: C, 62.03; H, 3.52; N, 19.95; S, 9.05.

#### 3.2.8. Synthesis of 2-[3-Imidoformic hydrazido-4-cyanopyrazol-2-yl]naphthalino[1,2-*d*]thiazole (**9**)

A mixture of compound **8** (0.01 mol), and hydrazine hydrate (0.01 mol) in acetic acid (20 mL) was refluxed 2 h. The obtained solid product was filtered off, dried and crystallized from methanol to give the title product **9** as a yellow powder. Yield: 62%; m.p.: 212–214 °C; IR (KBr, cm^−1^): 3420–3265 (NH, NH_2_) and 2215 (CN) cm^−1^; ^1^H-NMR (DMSO-*d*_6_): δ = 4.12 (s, 1H, CH=N), 6.12 (bs, 2H, NH_2_ exchangeable with D_2_O), 6.59–7.18 (m, 7H, 6Ar–H and 1H_Pyrazolo_) and 9.85 (b, 1H, NH exchangeable with D_2_O) ppm; ^13^C-NMR (DMSO-*d*_6_): δ = 111.2, 113.4, 116.9, 119.2, 120.6, 125.8, 127.5, 131.3, 133.3, 140.4 (Ar–C), 119.6 (C=N), 125.3 (CN), 136.2 (thiazole–C), 135.5, 139.3, 142.6 (pyrazole–C) ppm; MS *m*/*z* (%): 333 (M^+^, 21), 307 (34), 276 (29 ), 249 (37), 212 (22), 184 (100), 146 (45), 108 (19), 50 (26). Anal. Calcd. for C_16_H_11_N_7_S (333.41): C, 57.63; H, 3.32; N, 29.41; S, 9.62. Found: C, 57.48; H, 3.11; N, 29.16; S, 9.35.

#### 3.2.9. Synthesis of 2-[6-Methyl[1,2,4]triazolo[2,3-*c*]pyrimidino[6,5-*c*]pyrazol-2-yl]naphthalino[1,2-*d*]thiazole (**10**)

A suspension of compound **9** (0.01 mol) in a mixture of acetic acid/acetic anhydride (20 mL/5 mL) was heated under reflux for 2 h. After cooling, the reaction mixture poured into water, the precipitated product was filtered off, washed with water, dried and crystallized from ethanol to give the title product **10** as green powder. Yield: 68%; m.p.: 196–198 °C; ^1^H-NMR (DMSO-*d*_6_): δ = 2.15 (s, 3H, CH_3_), 4.23 (s, 1H, CH=N_Pyrazolo_), 6.59–7.18 (m, 7H, 6Ar–H, 1H_Pyrimidino_) ppm; ^13^C-NMR (DMSO-*d*_6_): δ = 21.5 (CH_3_), 112.9, 113.2, 116.2, 120.3, 122.2, 127.6, 130.7, 133.2, 138.2, 140.3 (Ar–C), 143.6, 145.5, 146.3, 148.5 (pyrimidine–C), 150.5 (triazole–C), 151.9 (pyrazole–C), 153.2 (thiazole–C) ppm; MS *m*/*z* (%): 357 (M^+^, 32), 342 (42), 316 (29), 276 (36), 249 (52), 225 (21), 184 (19), 158 (100), 108 (25), 85 (17). Anal. Calcd. for C_18_H_11_N_7_S (357.43): C, 60.48; H, 3.10; N, 27.43; S, 8.98. Found: C, 60.19; H, 2.87; N, 27.13; S, 8.70.

#### 3.2.10. Synthesis of 2-[7-Benzamido-6-iminopyrimidino[4,5-*c*]pyrazol-2-yl]naphthalino[1,2-*d*]thiazole (**11**)

A mixture of **8** (0.01 mol) and benzohydrazide (0.01 mol) in ethanol (30 mL) was refluxed 1 h. The formed solid was collected by filtration, dried and crystallized from dioxane to give the title product **11** as yellowish powder. Yield: 59%; m.p.: 276 °C; IR (KBr, cm^−1^): 3395–3240 (2NH) cm^−1^; ^1^H-NMR (DMSO-*d*_6_): δ = 5.37 (s, 1H, NH exchangeable with D_2_O), 6.59–7.18 (m, 13H, 11Ar-H, 1H_Pyrazolo_ and 1H_Pyrimidino_) and 9.31 (b, 1H, NH exchangeable with D_2_O) ppm; ^13^C-NMR (DMSO-*d*_6_): δ = 112.8, 114.6, 119.3, 120.7, 121.3, 123.2, 124.8, 128.2, 129.5, 131.4, 132.2, 135.3, 137.7, 138.2, 140.6, 142.3 (Ar–C), 144.6, 146.3, 149.5, 150.2 (pyrimidine–C), 151.4 (pyrazole–C), 152.2 (thiazole–C), 171.2 (C=O, amide) ppm; MS *m*/*z* (%): 437 (M^+^, 27), 422 (19), 345 (26), 317 (31), 261(53), 225 (29), 184 (36), 118 (100), 126 (32), 92 (17) 76 (24). Anal. Calcd. for C_23_H_15_N_7_SO (437.52): C, 63.13; H, 3.45; N, 22.41; S, 7.33. Found: C, 62.91; H, 3.18; N, 22.14; S, 7.15.

#### 3.2.11. Synthesis of 2-[3-(2-Ethoxy-4-oxo-5-dihydro-1,3-thiazol-3-yl)-4-cyanopyrazol-2-yl]-naphthalino[1,2-*d*]thiazole (**12**)

A mixture of compound **8** (0.01 mol) and mercaptoacetic acid (0.01 mol) in dry benzene (30 mL) was stirred under reflux for 3 h. After cooling, the obtained solid product was collected by filtration, washed with n-hexane, dried and crystallized from toluene to give the title product **12** as a yellow powder. Yield: 67%; m.p.: 193 °C; IR (KBr, cm^−1^): 2223 (CN) and 1680 (C=O amid) cm^−1^; ^1^H-NMR (DMSO-*d*_6_): δ = 1.14 (t, 3H, *J* = 6.05 Hz, CH_3_), 3.27 (s, 2H, CH_2_), 3.41 (q, 2H, *J* = 7.05 Hz, CH_2_), and 7.15–7.62 (m, 7H, 6Ar–H and1H_Pyrazolo_) ppm; ^13^C-NMR (DMSO-*d*_6_): δ = 22.5 (CH_3_), 57.3 (CH_2_), 110.8, 113.5, 116.4, 119.4, 120.6, 122.5, 126.2, 130.2, 132.3, 137.5 (Ar–C), 121.2 (CN), 144.2, 146.4, 151.1 (pyrazole–C), 145.2, 148.1, 150.3 (2 thiazole–C), 170.4 (C=O) ppm; MS *m*/*z* (%): 420 (M^+^, 36), 394 (21), 366 (42), 253 (31), 212 (28), 184 (100), 146 (37), 108 (25), 50 (19). Anal. Calcd. for C_20_H_14_N_5_S_2_O_2_ (420.56): C, 57.11; H, 3.35; N, 16.65; S, 7.61. Found: C, 56.87; H, 3.12; N, 16.39; S, 15.26.

#### 3.2.12. Synthesis of 2-[3-Ethoxy-5-dihydro-6H-7-oxo-[1,3]thiazolo[3,4-*b*]pyrimidino[4,5-*c*]pyrazol-2-yl]naphthalino[1,2-*d*]thiazole (**13**)

A suspension of compound **12** (0.01 mol) in sodium ethoxide (1.0 g sodium in 30 mL ethanol) was heated under reflux for 3 h. Excess of solvent was removed under reduced pressure to dryness, the residue was triturated with hot water. The formed solid was filtered off, washed with water, dried and crystallized from ethanol to give the title product **13** as a yellow powder. Yield: 72%; m.p.: 181 °C; IR (KBr, cm^−1^): 3362–3215 (NH) and 1692 (C=O amide); ^1^H-NMR (DMSO-*d*_6_): δ = 1.14 (t, 3H, *J* = 6.05 Hz, CH_3_), 3.27 (s, 2H, CH_2_), 3.41 (q, 2H, *J* = 7.05 Hz, CH_2_), and 7.15–7.62 (m, 7H, 6Ar–H and 1H_Pyrazolo_) and 10.12 (b, 1H, NH exchangeable with D_2_O) ppm; ^13^C-NMR (DMSO-*d*_6_): δ = 21.2 (CH_3_), 54.3 (CH_2_), 112.2, 114.3, 117.4, 118.3, 120.1, 124.3, 127.6, 130.8, 133.3, 139.3 (Ar–C), 144.6, 147.3, 151.6 (pyrimidine–C), 150.4 (pyrazole–C), 146.5, 148.9, 151.4 (2 thiazole–C), 169.1 (C=O, amide) ppm; MS *m*/*z* (%): 423 (M^+^, 28), 378 (52), 303 (19), 260 (22), 223 (41), 184 (100), 146 (37), 108 (28), 85 (35), 50 (19). Anal. Calcd. for C_20_H_17_N_5_S_2_O_2_ (423.40): C, 56.87; H, 4.05; N, 16.54; S, 15.12. Found: C, 56.65; H, 4.87; N, 16.35; S, 14.92.

### 3.3. Pharmacological Evaluation

#### 3.3.1. Animals

Albino rats (95) weighing 20–100 g were used and obtained from the Animal House Colony, Research Institute of Ophthalmology, Giza, Egypt. All animals (Guidelines for responsible conduct of research revised: march 2011 office of research integrity 132 cathedral of learning 412-624-3007) were maintained according to standard international human care and divided into fourteen equal groups. The animals were acclimatized for a period of two weeks in our laboratory environment prior to the study. They were housed in polypropylene cages, maintained under standard laboratory conditions, and fed with standard diet and water.

#### 3.3.2. Evaluation of Transcriptional Activity for Human Androgen Receptor

##### Establishment of Chinese Hamster Cell Model

Chinese Hamster Ovary (CHO) Cells Stably Transfected with Human Androgen Receptor Gene and Mouse Mammary Tumor Virus (MMTV)-Luciferase Reporter Gene or SV40-Luciferase Gene: CHO cells were maintained in alpha-modified Eagle’s medium supplemented with 10% fetal bovine serum (FBS). The culture medium of neomycin-resistant clone cells was supplemented with 10% dextran-coated charcoal-stripped FBS (DCC-FBS) and 500 mg/mL of neomycin. The CHO cells were transfected at 40%–70% confluence in 10-cm petri dishes with a total of 20 mg DNA (plasmide mammalian neow luciferase, (pMAMneoLUC)); Mouse mammary tumor virus (MMTV)-luciferase reporter plasmid and pSG5-hAR; human androgen receptor expression plasmid, or Simian vacuolating virus 40 (SV40-LUC); SV40-luciferase reporter plasmid containing neomycin resistant gene) by calcium phosphate mediated transfection. The stable transfected cells were selected in the culture medium supplemented with neomycin. The selected clone was designated as AR/CHO#3 (human AR gene and MMTV luciferase reporter gene integrated CHO cell) or SV/CHO#10 (SV-40-luciferase reporter gene integrated CHO cell), respectively [31].

##### Activities of the Tested Compounds to Inhibit Androgen Receptor Mediated Transcription Induced by Dihydrotestosterone (DHT) (AR Antagonistic Activity)

The stable transfected AR/CHO#3 or SV/CHO#10 cells were plated onto 96-well luminoplates (Packard, Palo Alto, CA, USA) at a density of 2 × 104 cells/well, respectively.

Four to eight hours later, the medium was changed to a medium containing DMSO, 0.3 nM of DHT, or 0.3 nM of DHT, and the tested compound. At the end of incubation, the medium was removed and the cells were lysed with 20 mL of lysis buffer (25 mM Tris–HCl (pH 7.8), 2 mM dithiothreitol, 2 mM 1,2-cyclo-hexanediamine-tetraacetic acid, 10% glycerol and 1% Triton X-100). Luciferase substrate (20 mM Tris–HCl (pH 7.8), 1.07 mM magnesium carbonate basic (MgCO_3_)_4_Mg(OH)_2_·5H_2_O, 2.67 mM MgSO_4_·7H_2_O, 0.1 mM ethylenediaminetetraacetic acid (EDTA), 33.3 mM dithiothreitol, 0.27 mM CoA, 0.47 mM luciferin, 0.53 mM ATP) was added, and luciferase activity was measured with a ML3000 luminometer (Dynatech Laboratories, El Paso, TX, UAS). AR antagonistic activities were calculated by the formula below:

AR antagonistic activity (%) = 100 (*I* − *X*) / (*I* − *B*) (1)

*I*: (luciferase activity of AR/CHO#3)/(luciferase activity of SV/CHO#10) in the presence of 0.3 nM of DHT, *B*: (luciferase activity of AR/CHO#3)/(luciferase activity of SV/CHO#10) in the presence of DMSO, *X*: (luciferase activity of AR/CHO#3)/(luciferase activity of SV/CHO#10) in the presence of 0.3 nM of DHT and the tested compound.

The concentration of compounds showing 50% AR antagonistic activity, or IC_50_ values, was obtained by nonlinear analysis using a statistical analysis system (SAS).

#### 3.3.3. *In Vivo* Evaluation of Anti-Androgenic Activities in Castrated Immature Rats

Androgen-treated male Wistar rats were obtained from the Animal House Colony, Research Institute of Ophthalmology, Giza, Egypt. Prepubertal male rats aged 3 weeks were castrated using the scrotal route under ether anesthesia. Three days after the castration, testosterone propionate (TP, 0.5 mg/kg, was administered subcutaneously (s.c.) once daily for 5 days, alone or in combination with the tested compound (10–30 mg/kg, *per os* (*p.o.*)). TP was dissolved in cotton seed oil containing 5% ethanol. The tested compound was suspended with 0.5% methylcellulose. The rats were sacrificed by excessive chloroform anesthesia 6 h after final dosing, and both their ventral prostates and seminal vesicles-coagulate glands were removed and weighed. The anti-androgenic activity was expressed as a percentage of inhibition of the TP effect (TP-treated rats were arbitrarily assigned a value of 0% and vehicle-treated rats a value of 100%) [31].

#### 3.3.4. Anti-Prostate Cancer Screening Anti-Androgenic Bioassay in Human Prostate Cancer Cells

Human prostate cancer LNCaP and PC-3 cells were maintained in RPMI medium and Dulbecco’s minimum essential medium (DMEM), respectively. Both media were supplemented with penicillin (25 units/mL), streptomycin (25 mg/mL), and 10% fetal calf serum. For the androgen receptor transactivation assay, an androgen-dependent reporter gene transcription test was employed as the primary screening for potential anti-androgen identification. This assay was first performed in LNCaP cells, which express a clinically relevant mutant AR. Once anti-androgenic activity was detected in the LNCaP AR transactivation assay, compounds were re-examined for their potential activity against wild type AR. Wild type AR transactivation assay was performed in PC-3 host cells, which lack an endogenous, functional AR. The method and conditions of cell and gene transfection have been described previously. In brief, cells were plated in 24-well tissue culture dishes for 24 (PC-3 cells) or 48 (LNCaP cells) h prior to transfection. Subsequently, LNCaP cells were transfected with a reporter gene, MMTV-luciferase, which contains a MMTV-long terminal repeat (LTR) promoter and an androgen receptor binding element, and Renilla Luciferase Control Reporter Vectors 40 (PRL-SV40), which served as an internal control for transfection efficiency. PC-3 cells were transfected with a wild type AR expression plasmid, plasmide gene-5 androgen recptor (pSG5AR), in addition to the above-mentioned MMTV-luciferase reporter gene and PRL-SV40 internal control. SuperFect (Qiagen, Chatsworth, CA, USA) was employed as the transfection reagent following the manufacturer’s recommendations. At the end of a 5-h transfection, the medium was changed to DMEM or RPMI supplemented with 10% charcoal dextran-stripped, *i.e.*, androgen-depleted, serum. After 24 h, the cells were treated with 1 nM of DHT and/or test compounds at the designated concentrations.

After 24 h the cells were harvested for luciferase activity assay using the Dual Luciferase Assay System (Promega, Madison, WI, USA.). The derived data was expressed as relative luciferase activity normalized to the internal luciferase control. Cells cultured in medium containing DHT (androgen), as a positive control, induced a marked increase in reporter gene expression. Test compounds capable of significantly suppressing this DHT induced reporter gene expression were identified as potential anti-androgens [32].

## 4. Structure Activity Relationship (SAR)

Polycyclic ring systems highly increase activity, as observed in the cases of compounds **10**, **12** and **13** that contain a polyhetrocyclic-fused ring system that provided potent activities.

Although compound **1** is a polyheterocyclic-fused ring system, it is completely free from any activity; this can lead to the conclusion that the pyrazoline ring moiety is essential for the activities.

A non-fused ring system, is present in compounds **12** and **9**, which are less active than their polyheterocyclic-fused ring system chemical alternate derivatives **10** and **13** respectively. Also the separated ring system of derivative **12** was the least active one, perhaps due to electronic clouds and charge separation.

The free hydrazyl moiety plays an important role in intensifying the activity probably due to their proton accepting and electron donating functions; compound **9** is more active than compound **8**.

Compound **4** that contains a bi-functional fused pyrazoline ring system is more active than those containing a pyrazoline-fused system with 6 member heterocyclic ring systems (compounds **3**, **5**, **6**, **7**, and **11**).

Regarding the fusion of 6 member heterocyclic ring systems (compounds **3**, **5**, **6**, **7** and **11**) the descending orders of potency were **3**, **11**, **5**, **6** and **7**; this led to the conclusion that the 6 member ring system containing two hetero atoms is more active than compounds containing one hetero-atom; compounds **3**, **5**, **6** and **11** are more active than compound **7**. Those containing two heteroatom’s with a nitrogen atom were more active than compounds containing sulfur one; compounds **3**, **5** and **11** more active than compound **6**.

## 5. Conclusions

The starting material **1** was used to synthesize the 3-amino-4-cyanopyrazolo derivative **2** which was treated with formamide or hydrazine hydrate to afford the corresponding aminopyrazolopyrimidine **3** and aminopyrazolopyrazole derivative **4**, respectively. Compound **2** was treated with ammonium isothiocyanate, carbon disulfide or ethyl acetoacetate to afford compounds **5**–**7**, respectively. Treatment of **2** with triethylorthoformate afforded derivative **8**, which was treated with hydrazine hydrate to give **9** followed by cyclized with acetic anhydride to **10**. Finally, compound **8** was reacted with benzoic acid hydrazide or mercapto acetic acid to give compounds **11** and **12**, respectively. Compound **12** was cyclized with sodium ethoxide to afford the pyrazolothiazolopyrimidine derivative **13**. The synthesized compounds were evaluated for their androgen receptor antagonists and anti-prostate cancer activities compared to that of Bicalutamide as a positive control. The acute toxicity of the compounds was assayed via the determination of their LD_50_. Most of the newly synthesized compounds were found to have potent AR antagonistic activities *in vitro* as compared with the reference drug Bicalutamide, while, compound **1** is devoid of any activity.
